# Assessment of drivers of *Listeria* environmental monitoring programs in small- and medium-sized dairy processing plants

**DOI:** 10.3168/jdsc.2024-0692

**Published:** 2025-03-18

**Authors:** Caroline Motzer, Aljosa Trmcic, Nicole Martin, Martin Wiedmann, Samantha Bolten

**Affiliations:** Milk Quality Improvement Program, Department of Food Science, Cornell University, Ithaca, NY 14853

## Abstract

Small- and medium-sized dairy processing plants (SMDPP) may face unique challenges, such as limited financial resources, when implementing *Listeria* environmental monitoring programs (EMP). To better understand what drives investment in *Listeria* EMP, 9 SMDPP, which had previously participated in an ∼1-yr longitudinal study focused on implementing *Listeria* EMP, completed a questionnaire regarding their EMP-associated costs. This questionnaire was used to obtain each plant's (1) self-reported product value (i.e., estimated total value of finished product [ETVFP] in a plant at any given time) and other EMP-associated costs (e.g., corrective action costs), as well as (2) perceived ability to control pathogens in their processing environment. Additionally, we used a predefined rubric to assess each SMDPP's commitment to their *Listeria* EMP; these data were used to obtain a “food safety culture score” out of 225 possible points. Overall, a wide range of total EMP investments ($1,186.96–$55,530.58) and food safety culture scores (56 out of 225 to 222 out of 225) were reported across plants. Linear regression analysis identified that predictor variables of plant size, overall environmental *Listeria* prevalence during the EMP implementation period, ETVFP, and food safety culture score were not significantly associated with the outcome of the total amount of money that plants spent on their *Listeria* EMP (i.e., their total EMP investment). Although small, our study suggests that SMDPP's financial investments in *Listeria* EMP may be influenced by other external factors or factors not related to food safety, such as owners' gut feelings, beliefs, and attitudes. To address this, we recommend that food safety training and educational programs for small and medium food businesses include coverage of food safety–related business risks.

## Highlights

• Nine dairy plants were evaluated for costs associated with their Listeria EMP.
• Total Listeria EMP investment varied widely across plants.
• Corrective actions accounted for the majority of total EMP investment for 7 of 9 plants.

## Graphical Abstract

**Figure:**
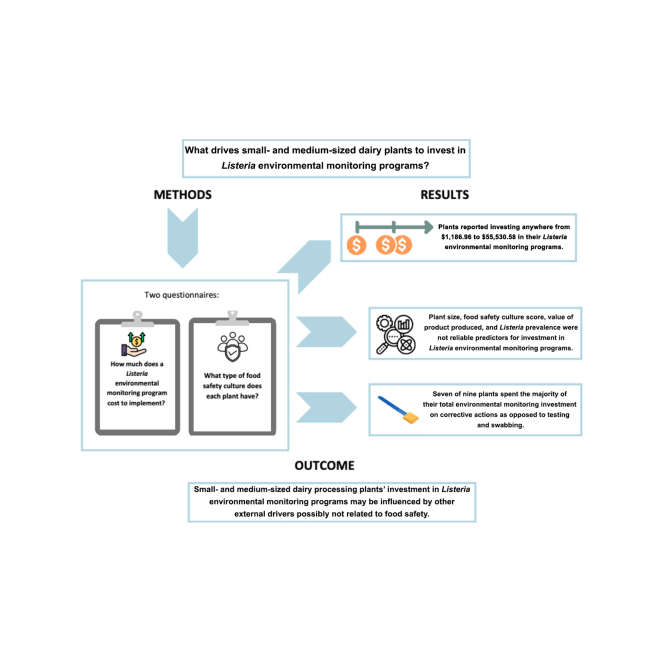
**Summary:** Small- and medium-sized dairy processing plants (SMDPP) may face unique challenges when implementing *Listeria* environmental monitoring programs (EMP). To understand their EMP investment decisions, we evaluated 9 SMDPP for (1) their food safety culture and commitment to *Listeria* EMP implementation and (2) financial costs associated with their Listeria EMP (e.g., costs of swabbing and testing, costs of corrective actions, and estimated values of the total finished product). Corrective actions accounted for the majority of total EMP investment in 7 plants, highlighting possible prioritization of corrective actions by SMDPP. A linear model identified that plant size, overall Listeria prevalence, estimated total value of the finished product, and food safety culture were not reliable predictors of total EMP investment, suggesting the influence of external factors that are potentially not related to food safety. Further evaluations of EMP investment drivers and food safety economics are needed to develop targeted strategies for food-safety support.

*Listeria monocytogenes* (**Lm**) is a foodborne pathogen that causes listeriosis, a bacterial infection that is associated with high mortality and hospitalization rates, making it a considerable public health concern (European Food Safety Authority, 2018). Importantly, Lm can persist in the environments of food processing plants, increasing the potential for contamination of ready-to-eat (**RTE**) foods for human consumption with Lm (Cartwright et al., 2013). To address this public health concern, regulations, third parties, and various internationally recognized food safety frameworks (e.g., Brand Reputation through Compliance Global Standard) have highlighted *Listeria* environmental monitoring programs (**EMP**) as a tool to better control Lm and other *Listeria* spp. in food processing environments based on risk (Zoellner et al., 2018; Brand Reputation through Compliance Global Standard, 2022). Notably, several recent listeriosis outbreaks and Lm recalls have implicated RTE dairy products manufactured by small- and medium-sized dairy processing plants (**SMDPP**) that either (1) did not have *Listeria* EMP in place (Centers for Disease Control and Prevention, 2008; US Food and Drug Administration, 2022) or (2) had *Listeria* EMP that were deemed ineffective at controlling Lm based on regulatory inspectional observations (U.S. Food and Drug Administration, 2017a, U.S. Food and Drug Administration, 2017b). This highlights the importance of continuing research geared toward implementation of proper *Listeria* EMP in SMDPP.

An important aspect of making food safety programs practical and enticing for businesses is to understand what drives a business to invest financial resources in food safety programs. Possible factors that might lead SMDPP to make insufficient investments in their *Listeria* EMP could be limited financial resources (Bhaskaran, 2006; Nayak and Waterson, 2017) or underappreciation of food safety programs due to poor food safety culture or limited education on *Listeria*-associated economic business risks (Yiannas, 2009; Belias et al., 2024). Currently, there is limited research characterizing the financial investments that smaller food businesses allocate toward their food safety programs, such as *Listeria* EMP, possibly due to the sensitive nature of financial data (Gomez and Marks, 2020). Moreover, although several studies have sought to assess the food safety culture of a variety of food business operations (Zanin et al., 2021), few have assessed food safety culture in smaller establishments (Nayak and Waterson, 2017), and none (to the authors' knowledge) have evaluated the relationship between food safety culture and financial investment in *Listeria* control programs for smaller establishments. Thus, the goals of this study were to (1) provide a benchmark for how much SMDPP are investing in *Listeria* EMP and (2) identify what drives SMDPP to invest in *Listeria* EMP. Toward this end, we evaluated 9 SMDPP, which had previously implemented *Listeria* EMP in an ∼1-yr longitudinal study (Bolten et al., 2024a,b), using 2 different questionnaires to assess both (1) their food safety culture and commitment to *Listeria* EMP implementation and (2) their financial investment in *Listeria* EMP. We hypothesized that facilities with larger product value (and therefore at greater risk of monetary losses due to *Listeria* contamination in finished products) and stronger food safety culture would show increased investment in their *Listeria* EMP.

The 9 SMDPP evaluated in this study are located in the northeastern United States and primarily manufacture either fluid milk (plants CL, N, and W), cheese (plants CM, BY, and CQ), or ice cream (plants CN, CO, and CP). Plants N and W were classified as medium-sized processing plants (i.e., processing between 453,592.37 and 45,359,237 kg of milk solids from raw milk per year), and the remaining 7 were classified as small-sized (i.e., processing <453,592.37 kg of milk solids from raw milk per year). All 9 SMDPP previously participated in an ∼1-yr longitudinal study focused on implementing and evaluating *Listeria* EMP for *Listeria* control. This involved collection and testing of environmental sponges for *Listeria* detection by the research team through both initial sampling events before *Listeria* EMP implementation and follow-up sampling events ∼1 yr after implementation as described by Bolten et al. (2024a) for 8 plants (i.e., plants CL, CM, CN, CO, CP, CQ, N, and W) and by Bolten et al. (2024b) for plant BY.

We collected information on the research team's perception of each SMDPP's food safety culture and overall dedication to *Listeria* EMP implementation in a questionnaire (questionnaire 1), which was administered to 5 members of the research team who worked with the SMDPP during the *Listeria* EMP implementation period, via a Qualtrics Survey (Qualtrics, Provo, UT) ∼1 mo after the longitudinal *Listeria* EMP study had concluded. Questionnaire 1 was composed of 9 descriptive statements pertaining to how proactive a given plant was at carrying out activities that are necessary for a successful *Listeria* EMP (Spanu and Jordan, 2020), including commitment to routine swabbing, root cause analysis, implementation of corrective actions, and the attitude of management and other employees toward food safety. For each plant, research team members were asked to rate their level of agreement with each of the 9 statements on a 5-point (1 = no control; 5 = full control) Likert scale (Likert, 1932). All research team member ratings across the 9 statements were summed to yield a “food safety culture score” of up to 225 possible points for each plant.

We also collected information on these plants' *Listeria* EMP financial investments and overall perception of pathogen control in a separate questionnaire (i.e., questionnaire 2), which was administered to the plants via email ∼1 yr after the longitudinal *Listeria* EMP study had concluded. Questionnaire 2 was composed of both short answer and multiple choice questions, where SMDPP were instructed to report several financial metrics (in US dollars), including the estimated cost of corrective actions during the 1-yr *Listeria* EMP implementation period and the estimated total value of finished product (**ETVFP**) that typically is in storage in the plant on any given day, as well as to rate on a scale of 1 to 5 their perceived control (i.e., 1 = no control, 2 = little control, 3 = some control, 4 = mostly under control, and 5 = full control) over pathogens in their plant after participating in the longitudinal *Listeria* EMP study. Participation in this questionnaire was voluntary and no compensation was provided. Both questionnaires, along with raw data, can be accessed in a publicly available repository (see Notes).

All data obtained from questionnaires was formatted and cleaned to facilitate statistical analysis. For example, if a plant reported a range of values for a given short answer question, the mean of the 2 values was used for analyses (e.g., if “$80,000–$100,000” was reported, $90,000 was used for analysis). In cases where a plant reported spending less than a given value for a given short answer question, a value of 1 unit lower than the reported value was used for analyses (e.g., if <$5,000 was reported, $4,999 was used for analysis). Estimated environmental swabbing and testing costs for each SMDPP were calculated by multiplying the number of environmental sponge samples that were collected and tested by individual plants during routine sampling events in the longitudinal *Listeria* EMP study (Bolten et al., 2024a, Bolten et al., 2024b) by the estimated cost of sampling materials (i.e., environmental sponge swabs, $1.50–$1.86, depending on sponge type) plus laboratory testing fees ($20.00 per swab). The value of “total EMP investment,” defined as the costs associated with a given plant's implementation of *Listeria* EMP over the ∼1-yr period, was then calculated for each plant by adding estimated swabbing and testing costs to the estimated cost of corrective actions (reported in the *Listeria* EMP-related costs questionnaire). In addition to questionnaire data obtained from the current study, we also used previously reported *Listeria* prevalence data from the 1-yr longitudinal *Listeria* EMP study (Bolten et al., 2024a,b) in statistical analyses to assess whether overall *Listeria* prevalence (the percent of environmental sponge samples that tested positive for *Listeria* across initial and follow-up sampling events) was associated with the outcome of “total *Listeria* EMP investment,” and whether changes in *Listeria* prevalence after 1 yr of EMP implementation (reported here as the percentage of *Listeria* positive samples in the follow-up sampling event − the percentage of *Listeria* positive samples in the initial sampling event in each plant) was influenced by the predictor of “investment in corrective actions,” and whether *Listeria* prevalence in the follow-up sampling event was correlated with plants' self-reported perceived control over pathogen presence at the end of the study period (Table 1).

**Table 1:** Environmental monitoring program (EMP)-associated costs, *Listeria* prevalences (Bolten et al., 2024a,b), food safety culture scores, and pathogen control perceptions reported for 9 small- and medium-sized dairy processing plants

Plant ID	ETVFP in $^3^	EMP-associated costs^1^			*Listeria* prevalence^2^			Food safety culture score (out of 225)^4^	Plants' perceived control over pathogens after *Listeria* EMP implementation (out of 5)^5^
		Total EMP investment in $	Corrective action cost in $ (% of total EMP investment)	Swab and testing cost in $	Initial and follow-up (%)	Follow-up (%)	∆ (percentage points)		
BY	10,000	25,903	25,000 (97)	903	29	8	−42	190	4
CL	20,000	2,215	2,000 (90)	215	28	25	−5	79	3
CM	100,000	5,796	<5,000 (86)	796	3	3	0	198	2
CN	100,000	5,215	5,000 (96)	215	39	15	−48	100	3
CO	7,500	1,389	980 (71)	409	15	20	10	122	4
CP	200,000	5,000	5,000 (100)	0	19	36	34	56	3
CQ	40,000	1,187	400 (34)	787	12	15	7	166	4
W	80,000–100,000	55,531	50,000 (90)	5,531	27	22	−9	129	4
N	2,200,000	12,485	200 (2)	12,285	3	6	5	222	4

1 Represents costs associated with a given plant's implementation of *Listeria monocytogenes* (Lm) EMP over the ~1-yr period. Total EMP investment was calculated as the sum of swab and testing costs and corrective action costs. Swab and testing costs were calculated by multiplying the number of environmental sponge swabs used by a given plant as part of their routine EMP sampling by the estimated costs of sampling materials (i.e., environmental sponge swabs, $1.50–$1.86, depending on sponge type) and laboratory testing fees ($20.00 per swab). Corrective action costs were self-reported by plants.

2 Refers to the percent of environmental sponge samples that tested positive for Lm across initial and follow-up sampling events. ∆ represents the change in Lm percent of positive environmental sponge samples in the follow-up sampling event compared with the initial sampling event. All Lm prevalence data were obtained in two previous studies; see Bolten et al. (2024a,b) for more details.

3 ETVFP = estimated total value of all finished product in a plant at any given time, self-reported by plants.

4 Indicates the research team's perception of each plant's dedication to EMP implementation and overall food safety culture. Higher scores indicate stronger EMP dedication and food safety culture.

5 Self-reported by plants. Each plant chose 1 of the following statements, each of which was associated with a point value: 1 = no control, 2 = little control, 3 = some control, 4 = mostly under control, and 5 = full control.

Statistical analysis was performed in R version 4.3.3 (https://www.r-project.org). A linear regression model was used to test the effect of ETVFP, annual kilograms of milk solids processed from raw milk, overall *Listeria* prevalence, and food safety culture score on the outcome of “total EMP investment” (i.e., the total dollar amount investment by a given plant in their *Listeria* EMP); total EMP investment values were log-transformed to satisfy normality assumptions. Additionally, a linear regression model was used to assess the effect of investments in corrective actions (i.e., the percentage of total EMP investment that was allocated specifically to corrective actions) on the outcome of “change in *Listeria* prevalence in the follow-up sampling event compared to the initial sampling event.” Finally, Pearson's product moment correlation test was conducted to compare plants' perceptions of their own pathogen control to follow-up *Listeria* prevalence. Alpha was set to 0.05 for all analyses to denote significant differences.

Results from questionnaires showed wide ranges in both ETVFP- and EMP-related costs.

The ETVFP values ranged from $7,500 to $2,200,000, with 7 of 9 plants reporting ETVFP values of ≤$100,000 (Table 1). Total EMP investment values ranged from $1,187 to $55,531, with the majority (6 of 9) of plants investing <$10,000 in their *Listeria* EMP. Finally, food safety culture scores ranged from 56 out of a possible 225 to 222 out of 225, with the majority (6 of 9) of plants receiving food safety culture scores between 100 and 198 out of 225.

Linear regression analysis showed that there was no significant association between the predictor variables of ETVFP, plant size (i.e., annual kilograms of milk solids processed from raw milk), overall *Listeria* prevalence, and food safety culture score on the outcome of “total EMP investment” (*P* = 0.39), indicating that none of these factors could represent reliable predictors for how much money SMDPP invest in their *Listeria* EMP. This could indicate that other variables, potentially ones that are less obviously linked to food safety, may be driving SMDPP financial decision making. Yapp and Fairman (2004), who conducted interviews with 50 small- and micro-food businesses, identified enforcement by regulators as a primary driver of food safety investments, but this was not measured in our study. Additionally, it is important to note that decision making in small- or medium-sized food businesses can oftentimes be solely driven by the beliefs and attitudes of a single owner (Bhaskaran, 2006), who may be more likely to rely on factors not related to food safety to drive decision making. For example, Danielson and Scott (2006) compiled survey data by the National Federation of Independent Business to analyze the strategies small businesses use to evaluate business financial expenditures and found that small businesses tend to use less sophisticated expenditure evaluation tools, more often relying heavily on owners' gut feelings to evaluate expenditures. Therefore, it is possible that similar variables such as gut feelings from owners and upper management could also represent drivers of *Listeria* EMP investment for some of the SMDPP examined here, which could result in negative public health consequences (e.g., recalls and outbreaks) and negative business impacts. To the second point, our data showed that SMDPP with larger ETVFP (and therefore larger exposure to monetary losses in the event of a *Listeria* issue that requires product destruction) do not seem to invest more into their EMP, which could suggest that SMDPP may not understand the business risks associated with *Listeria* recalls or outbreaks. To address this, food safety education materials targeting small- and medium-sized food businesses should include (1) an emphasis of economic business risks associated with *Listeria* and (2) an approach to quantify these risks. This education-focused strategy could be more effective for motivating individuals to invest more into *Listeria* control programs as opposed to traditional training that covers technical aspects of a *Listeria* EMP (e.g., how to pick sites, how to swab).

The SMDPP reported allocating varying percentages of their total EMP investment toward swabbing and testing versus corrective actions. For example, 7 of 9 plants (i.e., plants BY, CL, CM, CN, CO, CP, and W) spent more than 50% of total EMP investments on corrective actions, with plant CP reporting spending the highest percentage (100%), followed by plant BY (97%), and plant CN (96%; Table 1). Notably, the 3 plants that reported spending the lowest percentage of their total EMP investment on corrective actions (i.e., Plants N, CQ, and CO) had increases in *Listeria* prevalence in the follow-up sampling event compared with the initial sampling event (increases of 5, 7, and 10 percentage points in *Listeria* prevalence for plants N, CQ, and CO, respectively). This might anecdotally suggest that limited investments in corrective actions can have negative food safety outcomes (e.g., increased *Listeria* prevalence). However, linear regression analysis showed that, across all plants, there was no significant association (*P* = 0.32) between the percentage of total EMP investment that a given plant allocated toward corrective action costs and the change in *Listeria* prevalence in the follow-up sampling event compared with the initial sampling event. This could highlight that the effectiveness of a corrective action does not lie solely with its financial cost. Similarly, other studies reported that corrective actions may have limited effect on improvement of microbial food safety or quality related issues (Etter et al., 2017; Reichler et al., 2020). For example, Reichler et al. (2020) showed that interventions focused on improving employee training and modifying clean-in-place procedures were not able to significantly decrease presence of post-pasteurization contamination (**PPC**) in fluid milk produced by 4 large dairy plants in the northeast United States. Here, the authors postulated that possible reasons for the ineffectiveness of these interventions were lack of buy-in from management or misidentification of the true root cause of PPC contamination, which represent similar problems that could have affected the effectiveness of corrective actions carried out by the SMDPP in our study.

Interestingly, some responses from the EMP-related costs questionnaire revealed that select SMDPP may have inaccurate perceptions of their own pathogen control. For example, the plant that received the lowest food safety culture score (as determined by the research team's evaluation of each plant in questionnaire 1) and showed the highest *Listeria* prevalence in the follow-up sampling event (i.e., plant CP) reported their perceived control over pathogens after ∼1 yr of *Listeria* EMP implementation as “mostly under control” in questionnaire 2, which was the same response reported by the plant that received the highest food safety culture score and showed the lowest *Listeria* prevalence in the follow-up sampling event (i.e., plant N; Table 1). Moreover, a Pearson's coefficient correlation test comparing plants' perception of their own pathogen control to *Listeria* prevalence observed in each plant's follow-up sampling event identified a correlation coefficient of −0.31, potentially indicating plants' having inaccurate perceptions of their own pathogen control; however, this result was not significant (*P* = 0.41). Given that the 95% CI for this test was so large (i.e., −0.80 to 0.45), the lack of significance could be due to the limited sample size, which highlights a key limitation that should be considered when interpreting this and other statistical analysis results from this study. Furthermore, other factors, such as over-reporting of socially desirable values (e.g., overstating corrective action investments to appear responsible) should also be considered when interpreting this data. For example, plant CP reported spending $5,000 on *Listeria* EMP-related corrective actions during the 1-yr EMP implementation period, despite receiving the lowest food safety culture score of the 9 plants (56 out of 225) and not performing any environmental swabbing throughout the 1-yr study period (Bolten et al. 2024a), which may suggest their possible over-reporting of this particular value. Studies identifying reporting bias in favor of social norms in food safety contexts are not uncommon (Ungku Fatimah et al., 2014; da Cunha et al., 2019). For instance, da Cunha et al. (2019) created 2 structural equation models based on data collected from 183 food handlers in Brazil, one that represented data on food safety practices self-reported by food establishments and another that represented data on those same food safety practices observed by outside food safety inspectors. Findings showed a weak correlation (r = 0.33, *P* = 0.002) between the 2 models, indicating a discrepancy between self-reported and observed food safety behaviors, with the self-reported behaviors seeming to be overinflated compared with their observed behaviors. It is possible, that should SMDPP have more access to targeted education materials that address key business risks associated with *Listeria,* then SMDPP may feel empowered to improve their food safety programs and not feel the need to potentially over-report.

Overall, specific drivers of SMDPP investment in *Listeria* EMP could not be identified in this small study, and it is possible that small and medium food business owners do not use data-driven approaches to determine their investment into food safety programs. Additionally, although SMDPP seem to prioritize corrective actions, the effectiveness of these corrective actions may be associated with other variables, such as buy-in from management or proper identification of root causes. Finally, this study can serve as a foundation for further research regarding economic investment in food safety or food safety culture, and in doing so, results may help better tailor food safety education materials, especially ones focused on framing investment into *Listeria* EMPs as a business or enterprise risk-management strategy.

## References

[bib1] Belias A., Bolten S., Wiedmann M. (2024). Challenges and opportunities for risk- and systems-based control of *Listeria monocytogenes* transmission through food. Compr. Rev. Food Sci. Food Saf..

[bib2] Bhaskaran S. (2006). Incremental innovation and business performance: Small and medium-size food enterprises in a concentrated industry environment. J. Small Bus. Manag..

[bib3] Bolten S., Lott T.T., Ralyea R.D., Gianforte A., Trmcic A., Orsi R.H., Martin N.H., Wiedmann M. (2024). Intensive environmental sampling and whole genome sequence-based characterization of *Listeria* in small- and medium-sized dairy facilities reveal opportunities for simplified and size-appropriate environmental monitoring strategies. J. Food Prot..

[bib4] Bolten S., Ralyea R.D., Lott T.T., Orsi R.H., Martin N.H., Wiedmann M., Trmcic A. (2024). Utilizing whole genome sequencing to characterize *Listeria* spp. persistence and transmission patterns in a farmstead dairy processing facility and its associated farm environment. J. Dairy Sci..

[bib5] Brand Reputation through Compliance Global Standard (2022). Global Standard Food Safety (Issue 9). https://www.brcgs.com/product/global-standard-food-safety-issue-9/p-13279/.

[bib6] Cartwright E.J., Jackson K.A., Johnson S.D., Graves L.M., Silk B.J., Mahon B.E. (2013). Listeriosis outbreaks and associated food vehicles, United States, 1998–2008. Emerg. Infect. Dis..

[bib7] Centers for Disease Control and Prevention (2008). Outbreak of *Listeria monocytogenes* infections associated with pasteurized milk from a local dairy–Massachusetts, 2007. Morb. Mortal. Wkly. Rep..

[bib8] da Cunha D.T., de Rosso V.V., Pereira M.B., Stedefeldt E. (2019). The differences between observed and self-reported food safety practices: A study with food handlers using structural equation modeling. Food Res. Int..

[bib9] Danielson M.G., Scott J.A. (2006). The capital budgeting decisions of small businesses. J. Appl. Finance.

[bib10] Etter A.J., Hammons S.R., Roof S., Simmons C., Wu T., Cook P.W., Katubig A., Stasiewicz M.J., Wright E., Warchocki S., Hollingworth J., Thesmar H.S., Ibrahim S.A., Wiedmann M., Oliver H.F. (2017). Enhanced sanitation standard operating procedures have limited impact on *Listeria monocytogenes* prevalence in retail delis. J. Food Prot..

[bib11] European Food Safety Authority (2018). *Listeria monocytogenes* contamination of ready-to-eat foods and the risk for human health in the EU. EFSA J..

[bib12] Gomez C.B., Marks B.P. (2020). Monetizing the impact of food safety recalls on the low-moisture food industry. J. Food Prot..

[bib13] Likert R. (1932). A technique for the measurement of attitudes. Arch. Psychol..

[bib14] Nayak R., Waterson P. (2017). The assessment of food safety culture: An investigation of current challenges, barriers and future opportunities within the food industry. Food Control.

[bib15] Reichler S.J., Murphy S.I., Erickson A.W., Martin N.H., Snyder A.B., Wiedmann M. (2020). Interventions designed to control postpasteurization contamination in high-temperature, short-time-pasteurized fluid milk processing facilities: A case study on the effect of employee training, clean-in-place chemical modification, and preventive maintenance programs. J. Dairy Sci..

[bib16] Spanu C., Jordan K. (2020). *Listeria monocytogenes* environmental sampling program in ready-to-eat processing facilities: A practical approach. Compr. Rev. Food Sci. Food Saf..

[bib17] Ungku Fatimah U.Z.A., Strohbehn C.H., Arendt S.W. (2014). An empirical investigation of food safety culture in onsite foodservice operations. Food Control.

[bib18] U.S. Food and Drug Administration (2017). Vulto creamery issues voluntary recall of all soft, wash-rind raw milk cheeses because of possible health risk. https://www.fda.gov/safety/recalls-market-withdrawals-safety-alerts/vulto-creamery-issues-voluntary-recall-all-soft-wash-rind-raw-milk-cheeses-because-possible-health.

[bib19] U.S. Food and Drug Administration (2017). FDA Form 483: Vulto Creamery, LLC, Walton, NY. https://www.fda.gov/files/about%20fda/published/Vulto-Creamery–LLC–Walton–NY-483-Issued-03-22-2017.pdf.

[bib20] U.S. Food and Drug Administration (2022). Warning Letter: Big Olaf Creamery LLC. https://www.fda.gov/inspections-compliance-enforcement-and-criminal-investigations/warning-letters/big-olaf-creamery-llc-dba-big-olaf-642758-12092022.

[bib21] Yapp C., Fairman R. (2004). Compliance with food safety legislation in small and micro-businesses: Enforcement as an external motivator. J. Environ. Health Res..

[bib22] Yiannas F. (2009). https://doi.org/10.1007/978-0-387-72867-4.

[bib23] Zanin L.M., Stedefeldt E., Luning P.A. (2021). The evolvement of food safety culture assessment: A mixed-methods systematic review. Trends Food Sci. Technol..

[bib24] Zoellner C., Ceres K., Ghezzi-Kopel K., Wiedmann M., Ivanek R. (2018). Design elements of *Listeria* environmental monitoring programs in food processing facilities: A scoping review of research and guidance materials. Compr. Rev. Food Sci. Food Saf..

